# Development and inter-rater reliability of a simple prehospital mobility score for use in emergency patients

**DOI:** 10.1186/s12873-024-00944-9

**Published:** 2024-02-15

**Authors:** Søren Westh Asmussen, Jacob Metze Holme, Kurt Joensen, Stine Ibsen, Henrik Bøggild, Erika Frischknecht Christensen, Tim Alex Lindskou

**Affiliations:** 1 Centre for Prehospital and Emergency Research, Aalborg University Hospital, Aalborg University, Selma Lagerløfs Vej 249, Room 11.03.049, Gistrup, 9260 Denmark; 2 Centre for Prehospital and Emergency Research, Aalborg University Hospital, Aalborg University, Aalborg, Denmark; 3 Centre for Prehospital and Emergency Research, Aalborg University Hospital, Aalborg, Denmark; 4 Department of Physiotherapy, University College of Northern Denmark (UCN), Aalborg, Denmark; 5 Department of Health Science and Technology, Public Health and Epidemiology, Faculty of Medicine, Aalborg and Unit of Clinical Biostatistics, Aalborg University, Aalborg University, Aalborg University Hospital, Aalborg, Denmark; 6 Department of Clinical Medicine, Centre for Prehospital and Emergency Research, , Aalborg University Hospital, Aalborg University, Aalborg, Denmark; 7 Prehospital Emergency Services, North Denmark Region, Aalborg, Denmark

**Keywords:** Mobility, Mobility score, Prehospital, Emergencies, Clinical assessment, Ambulances, Early warning score, Reliability, Emergency medical service

## Abstract

**Background:**

Mobility assessment enhances the ability of vital sign-based early warning scores to predict risk. Currently mobility is not routinely assessed in a standardized manner in Denmark during the ambulance transfer of unselected emergency patients. The aim of this study was to develop and test the inter-rater reliability of a simple prehospital mobility score for pre-hospital use in ambulances and to test its inter-rater reliability.

**Method:**

Following a pilot study, we developed a 4-level prehospital mobility score based of the question”How much help did the patient need to be mobilized to the ambulance trolley”. Possible scores were no-, a little-, moderate-, and a lot of help. A cross-sectional study of inter-rater agreement among ambulance personnel was then carried out. Paramedics on ambulance runs in the North- and Central Denmark Region, as well as The Fareoe Islands, were included as a convenience sample between July 2020—May 2021. The simple prehospital mobility score was tested, both by the paramedics in the ambulance and by an additional observer. The study outcomes were inter-rater agreements by weighted kappa between the paramedics and between observers and paramedics.

**Results:**

We included 251 mobility assessments where the patient mobility was scored. Paramedics agreed on the mobility score for 202 patients (80,5%). For 47 (18.7%), there was a deviation of one between scores, in two (< 1%) there was a deviation of two and none had a deviation of three (Table 1).

Inter-rater agreement between paramedics in all three regions showed a kappa-coefficient of 0.84 (CI 95%: 0.79;0.88). Between observers and paramedics in North Denmark Region and Faroe Islands the kappa-coefficient was 0.82 (CI 95%: 0.77;0.86).

**Conclusion:**

We developed a simple prehospital mobility score, which was feasible in a prehospital setting and with a high inter-rater agreement between paramedics and observers.

**Supplementary Information:**

The online version contains supplementary material available at 10.1186/s12873-024-00944-9.

## Background

Early risk assessment of acutely ill patients is important to provide the right treatment at the right time. Risk assessment in the acute care setting is usually based on the patient’s complaints and alterations in vital signs, e.g., heart rate, blood pressure and oxygen saturation [1]. However, acutely ill patients who suffer a fatal outcome may present themselves with vital signs close to or within the normal range [2, 3]. Therefore, other risk factors are included in risk stratification, such as older age, co-morbidity and/or physiological capacity, such as mobility. A previous retrospective multicentre study shows that impaired mobility on presentation in the acute care setting is a more powerful predictor of 30-day mortality than age and comorbidity [4]. However, a limitation of the study was the use of different mobility assessments and that it was based on retrospective chart review, such as the patient’s ability to get on the bed unaided or to stand unaided. Studies of acutely ill patients’ mobility based on various methods such as lack of stable independent gait [5], the inability to stand or walk [6] or fast declining gait speed [7] indicate reduced mobility may be associated with mortality independently of traditional vital signs, age, and co-morbidities. Many acutely ill patients’ first meeting with the health services is pre-hospital in the ambulance service, with paramedics examining the patients acutely. Measurement of mobility in the ambulance before the initial treatment may potentially provide a clearer view of the patient's status and risk of deterioration and thereby improve risk assessment as early treatment in the ambulance is carried out on scene or en route to hospital. The treatment (such as pain relief, fluid resuscitation and immobilisation) given on scene and in the ambulance may interfere with the patients’ mobility, masking their initial condition. Early prehospital mobility scoring may give better and more valid estimations of the mobility of acute patients conveyed by ambulance. When patients are presenting at emergency departments it is difficult for the staff to estimate prior mobility, as this might have changed. Without a prehospital mobility assessment, the hospital staff do not have an impression the patient’s first mobility before treatment was commenced. To be feasible in the prehospital setting, mobility assessment should be a standardized procedure, simple to perform and reliable. Current mobility scores are complex and designed for in-hospital use and thereby might not be feasible in a prehospital setting.

## Methods

### Aim

The aim of this study is to develop a simple prehospital mobility score and to investigate the inter-rater reliability.

### Study design

Inter-rater reliability study in the North- and Central Denmark Region as well as the Faroe Islands. The study is reported according to the Guidelines for Reporting Reliability and Agreement Studies (GRRAS) [8].

### Setting

The study took place in two of the five administrative Regions of Denmark, specifically the North Denmark Region (citizens = 589.936), the Central Denmark Region (citizens = 1.326.340) [9], and the Faroe Islands (citizens = 52.619) [10]. In Denmark, each ambulance is operated by a crew of two ambulance professionals, of which at least one is at paramedic level (from heron all ambulance professionals are referred to as paramedics). Ambulances are dispatched by Emergency Medical Coordination Centres located in each region. The Emergency Medical Coordination Centre is manned by healthcare professionals answering calls from the emergency telephone line 1–1-2. The call takers assess the level of urgency and the main reason for calling, whereas technical dispatchers coordinate and dispatch ambulances [11]. The Faroe Islands are an autonomous region in the Kingdom of Denmark (also including Greenland). It is an archipelago in the North Atlantic Ocean and encompass more rural and rugged terrain compared to Denmark. The Faroese national ambulance service is part of the public sector and medical dispatching and staffing of ambulances are identical to Denmark [12].

### Pilot study

We initially developed a prehospital mobility score intended to use in prehospital conditions, applicable across different patient groups and not requiring extra equipment in the ambulance. We decided to base the mobility score on an assessment of the patient’s mobility as they were mobilized onto the ambulance trolley, with the use of the following question: “How much help did the patient need to be mobilized to the ambulance trolley” with the possible answers “no help”, “some help”, “moderate help” or “a lot of help. No help was described as walking without aid and thereby mobilizing to the trolley without help. Some help comprised patients receiving a helping hand to get onto the trolley thus the patients could help with mobilization to the trolley at this point. Moderate help was described as substantial support to get mobilized onto the trolley where the patient could help with mobilization to the trolley but to a limited extent. Lastly a lot of help was described as must be carried onto the trolley and the patient could not help with mobilization. At first, this version of the score was presented to a group of paramedics to find out if it was comprehensible, which resulted in one of the options for the questions being modified, from *some help* to *a little help.* The modified mobility score was tested in a pilot study, including a convenience sample of 80 unselected ambulance runs in January and February 2020. The two paramedics present in the ambulance were asked to score the patient individually according to the possible answers above. This was done by an observer present in the ambulance, who first asked one of the paramedics to score the patient, and then later the second paramedic, without them hearing each others answer.

Only the patients need for mobilization to the ambulance trolley were assessed, and daily life mobility was not collected. We found an unweighted agreement of 82%, and by conducting individual semi-structured interviews, identified the statement a little help and moderate help as being the ones most disagreed upon. All interviewees considered the question to be easily understandable and applicable (Supplementary file 1). After this development process, we conducted a large inter-rater reliability study.

### Rater, observer and subject population

The raters were paramedics and observers. The selection of paramedics was performed at random by the superior at the regional ambulance service or private providers and included staff on frontline ambulances as these handle emergency calls. Paramedics were informed by the Emergency Medical Services that observers could be present, and ask them to rate the patients mobility. As such the paramedics were included based on the observers availability. The second author also worked in the Central Denmark region as a paramedic.

For each ambulance run, two paramedics and one observer, present in the ambulance, scored the patients level of mobility. The observers checked that the data were collected and were also raters as they independently assessed the patient. The observers were the first, second and third authors, who were medical students and two army medics.

The subject population was a convenience sample of the ambulance patients whom the observers and paramedics were dispatched to. Only patients who were mobilized on the ambulance trolley were included. This includes medical, surgical, and trauma patients. Patients who were transported by chair or wheelchair users and thus not mobilized to the ambulance trolley were excluded by the observer. Patients of age 6 or younger were excluded due to compliance and/or possible ethical issues when mobilizing younger patients without their parents’ involvement. No patient data was included in the study, only paramedic and observers assessment of patient mobility. As such no identifiable patient information was collected.

### Rating process

The observer independently scored the patients' mobility level when they were mobilized to the trolley. The two paramedics individually scored the patients’ mobility level in the same way and reported it to the observer just after the patients’ handover to the hospital. The mobility score was noted on paper by the observer. Thereby paramedics were blinded to each other’s and the observer's ratings. They were instructed not to discuss patient mobility before handover and scoring at the hospital. This was ensured by the observer. The paramedics were asked the same way as in the pilot study. No training was given before the study start. Only ambulance runs, the region the data was collected, and the scores by the paramedics and observers were collected.

Due to COVID-19, an observer in the ambulances was not permitted in the Central Denmark Region, thus data collection was limited to two raters for this Region.

### Statistics

To assess inter-rater agreement between the two paramedics, a weighted Cohen’s kappa analysis with 95% confidence interval (95% CI) was estimated. The weighted analysis was linear with 1.00 unit for agreement, 0.66 unit for one deviation, 0.33 unit for two deviations and 0.00 unit for three deviations.

Likewise, when including the observer, a 3-raters weighted kappa analysis was performed. Kappa-coefficients (kappa) above 0.75 were evaluated as high agreement [13].

All statistics were carried out in STATA 16 (Statacorp, Texas) with the plugin “kappaetc” (Daniel Klein, Universität Kassel).

## Results

**Fig. 1:**
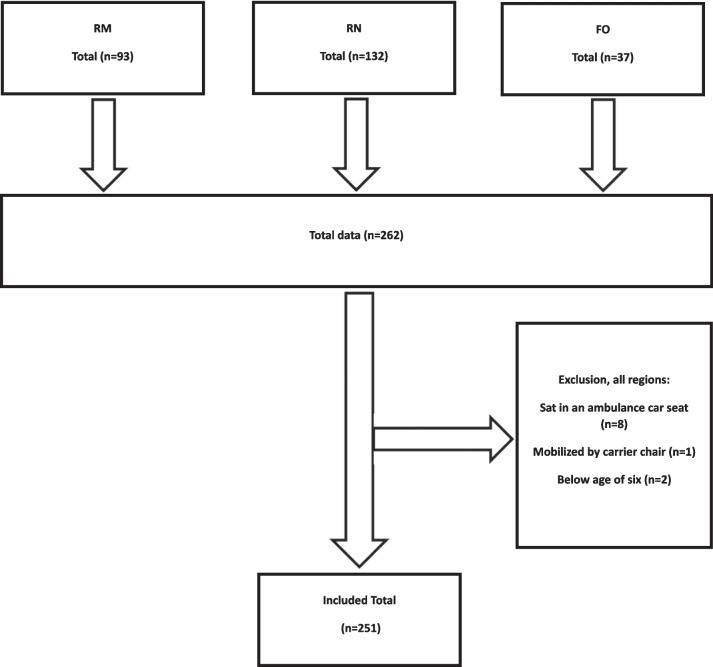
Flowchart Participants Main Data Collection. The data was collected from July 2020 to May 2021 from 07:00–22:00 in The Central Denmark Region (RM), The North Denmark Region (RN) and The Faroe Islands (FO)

We included the mobility assessment of 251 patients in the study. Of these 93 were from the Central Denmark Region, 130 were from the North Denmark Region and 28 were from the Faroe Islands. 11 patients were excluded as they were below the age of 6, transported by carrier chair or sat in the ambulance chair (Fig. 1).

Overall, for 202 (80.5%) of the patients, the paramedics agreed on their scores. The option most agreed on was “no help”, followed by “a little help”, “a lot of help” and “moderate help”. For 47 (18.7%) of the patients, there was a deviation of one between scores, in two (< 1%) of the patients there was a deviation of two and none had a deviation of three (Table 1).

**Table 1:**
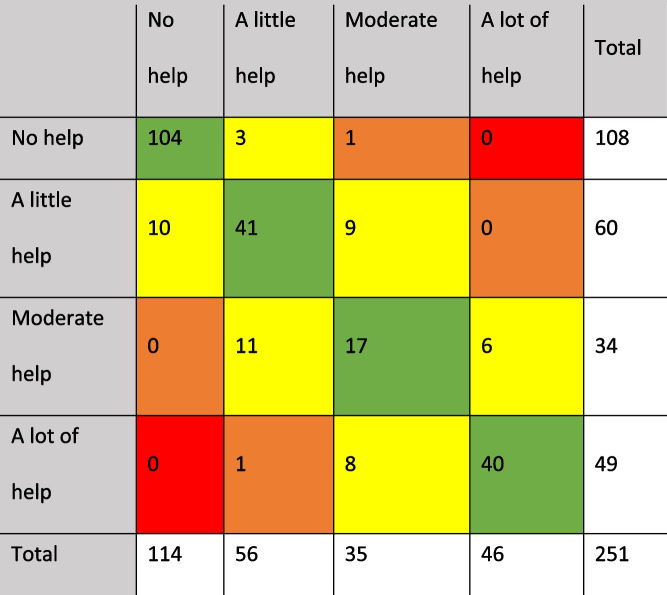
Distribution of mobility scores for the two paramedics. Green = agreement. Yellow = disagreement one deviation. Orange = disagreement two deviations.  Red = disagreement three deviations

### Estimates of reliability

Overall, inter-rater reliability among paramedics had a kappa estimate of 0.84 (95%CI 0.79;0.88) and a weighted agreement of 93.2%. Interrater reliability among paramedics and observers (the North Denmark Region and the Faroe Islands combined) had a kappa estimate of 0.82 (95%CI 0.77;0.86) with a weighted agreement of 92.1% (Table 2).

**Table 2:** Kappa estimates, 95% confidence interval (95% CI), per cent of weighted agreement for two raters (paramedics) and paramedics and research assistants (three raters)

Paramedics (2 raters)	Kappa estimate	95% CI	Weighted agreement (%)
All *(n* = *251)*	0.84	0.79; 0.88	93
North Denmark Region *(n* = *130)*	0.83	0.77; 0.89	93
Faroe Islands *(n* = *28)*	0.73	0.56; 0.89	88
Central Denmark Region *(n* = *93)*	0.87	0.80; 0.93	95
Paramedics and observer (3 raters)	Kappa estimate	95% CI	Weighted agreement (%)
All*(n = *158)*	0.82	0.77; 0.86	92
North Denmark Region *(n* = *130)*	0.82	0.77; 0.87	93
Faroe Islands *(n* = *28)*	0.76	0.62; 0.90	90

*Due to COVID-19 no third rater was allowed in the Central Denmark Region

## Discussion

We developed a simple mobility score, which was found feasible in a prehospital setting. It was found usable, understandable, and applicable by paramedics. Reliability of the prehospital mobility score in daily clinical use in the ambulances showed very high inter-rater agreement with weighted kappa above 0.90 between paramedics and between paramedics and observers. The similar agreement between the paramedics and the observer suggests that the evaluation could be carried out during everyday functions without hampering reliability.

A few studies have investigated mobility for risk assessment in emergency patients, and similarly to our study, they used simple items, such as lack of a stable independent gait [5] or the inability to stand or walk [6]. However, these studies concerned in-hospital patients, in the emergency department, the study populations were, smaller and the studies did not include reliability testing.

Most studies of mobility scores concern specific patient groups and show moderate to high reliability. A Dutch study investigated inter-rater reliability of a mobility measurement in a hospital setting for internal medicine and urology patients above the age of 18 at admission (The Activity Measure for post-acute care “6 click” Basic Mobility), which contains six items: rolling in bed, transfers in bed, transfer out of bed, standing, walking and climbing stairs. These activities are scored on a score from 1 (total assistance required) to 4 (no assistance required). The study showed weighted Kappa ranging from 0.65 to 0.84 for the six items [14]. The “6-click basic” mobility score compared to our score included elements such as climbing stairs. This is difficult to assess in a prehospital setting and excess categories can be complicated to use as the time is limited and the priority is to get the patient to the hospital.

Another study tested inter-rater reliability of mobility scores in an intensive care unit and one of them found a kappa of 0.80 based on five categories such as no activity, passive movement, sitting in bed, standing and ambulation [15]. Their 5-category mobility score showed high level of agreement similar to our study, but the categories were designed for an intensive care unit and specially trained intensive personal with a very sick and specific patient population that is hard to generalize upon compared to our broad prehospital population. A Canadian study tested inter-rater reliability of a mobility score based on six categories of intensive care patients with cardiovascular diseases. Their score showed a good inter-rater agreement kappa of 0.71 at admission [16]. This study provided an agreement that was lower than our 4-level inter-rater agreement kappa of 0,84. An Austrian study tested the reliability of a mobility score for in-hospital patients with musculoskeletal injuries. Their mobility score comprised 6 categories (BMS) ranging from change of position while lying in bed to climbing stairs. Their study showed a high inter-rater agreement with an intra-class correlation coefficient of 0.85 [17].

A small study from Houston, TX, used the Perme ICU mobility score comprising 15 different categories with 9 of them related to mobility. For the mobility categories the study found weighted kappa estimates ranging between 0.60 and 1.00 [18].

Although the studies mentioned comprised more categories, differ in settings, size, measured outcomes, and study population, they showed a level of moderate to high agreement compared to our high agreement based on a broader and diverse prehospital population. This suggests that ambulance paramedics quickly and reliable can evaluate the mobility of the pre-hospital patient using our score.

The score was tested in the prehospital setting during ambulance professionals’ routine work. It was then adapted based on user inputs. However, the current study did not investigate the validity, e.g. criterion, content or construct validity of the score, as the main focus was the development of a pragmatic score, and assessment of its reliability.

In the Central Denmark Region, it was not possible to include an observer due to COVID-19. The second main author also operated as a paramedic in this region and provided mobility scores. However, to address this, the second author provided his assessment first, before obtaining the second paramedics individual assessment. The effect of this limitation is therefore deemed minimal.

The Faroe Islands had a lower kappa value of 0,76 for paramedics and observers and was by far the smallest contributor to our data set with just 28 patients but also had the smallest population. The broad confidence interval suggests that the estimate might be the same as in the other regions (0.62;0.90). We included the Faroe Islands to test our mobility score on a wider range of patients.

The time delay between the observer's score on the scene and the paramedics reporting of the score after handover to a hospital may have introduced misclassification as they had to recall how the patient’s mobility was when taken onto the trolley. This misclassification could introduce both an over-and underestimation when interpreting the results but is likely nondifferential. This is also supported by the kappa estimates between paramedics and observers being similar.

The mobility score was tested in a daily prehospital setting on a broad, unselected subject population, thus putting its evaluation as close to the real use as possible. This was done to resemble the wide prehospital patient population reflecting that the score should encompass this variety.

No data on patients’ demographic characteristics or injury subgroups were collected. This limits the possibility to compare the patients between regions and their injury groups. The development of this simple prehospital mobility score and the high reliability forms the basis for future research including patient data and outcomes. The perspective is to perform studies about this score’s ability to stratify prehospital patients into risk groups and predict outcome, alone and in combination with early warning scores based on vital signs.

## Conclusion

We developed a simple mobility score feasible to use in the prehospital setting and with a very high level of inter-rater reliability. As such the score can provide a mobility score that is uniformly assessed by the paramedics. Next steps include investigating the association between the simple prehospital mobility score and patient outcome.

### Supplementary Information

**Additional file 1.** Interviews pilotstudy transcription.

## Data Availability

Data of the mobility scores are kept by the Centre for Prehospital and Emergency Research, Aalborg University Hospital and can be obtained by contacting Søren W. Asmussen by mail s.asmussen@rn.dk.

## Abbreviations

FO: The Faroe Islands
GRASS: Guidelines for Reporting Reliability and Agreement Studies
ICU: Intensive care unit
RM: The Central Denmark Region
RN: The North Denmark Region
