# Supplementary material for: Development and inter-rater reliability of a simple prehospital mobility score for use in emergency patients
Source: BMC Emerg Med. 2024 Feb 15;24:27. doi: 10.1186/s12873-024-00944-9 (PMC10868046; doi:10.1186/s12873-024-00944-9)
Supplement: Supplementary file 1 — Additional file 1. Interviews pilotstudy transcription. [file 12873_2024_944_MOESM1_ESM.pdf]

Black is for interviewer; red is for the interviewed paramedic.

Interviewer: Rikke

Interview one, date 14/2-2020, time 08:56

Position: Paramedic

Experience: 11 years

" What is your understanding of the mobility question: How much help did the patient need to be mobilized to the ambulance trolley?"

"What the question means?"

"Yes and if you think that it is relevant to investigate, and if the different answers categories are understandable?

Yes I understand that completely. I think that it is very relevant because we sometimes have patients that have no mobility at all In the acute fase, but in general our patients often are able to walk themselves to the ambulance trolley."

"And in relation to their mobility and mortality in the hospital, that that is what we want to investigate. There are studies that have shown that there is a correlation between low mobility when you arrive, and a worse outcome when you leave the hospital.

"Yes okay."

"But is has been small studies that has been made so far. So now we will try to make some here in June. I think it will be a large national study on 20 emergency departments in Denmark."

Interview two, date 14/2-2020, time 09:34

Position: Paramedic

Experience: 14,5 years

" What is your understanding of the mobility question: How much help did the patient need to be mobilized to the ambulance trolley?"

"I think that it is easy to understand and I understood the question."

" Okay, nothing was unclear?"

"It is not important with futher instruction"

"No? that is super."

Interview three, date 14/2-2020, time 11:18

Position: Assistant paramedic

Experience: 3 years

“ What is your understanding of the mobility question: How much help did the patient need to be mobilized to the ambulance trolley?”

“My understanding of it?”

Yes and if you think that it requires further instruction, or if it made sense?

“It made sense”.

“Okay.”

Interview four, date 14/2-2020, time 11:41

Position: Trainee

Experience: 3 years

“ What is your understanding of the mobility question: How much help did the patient need to be mobilized to the ambulance trolley?”

“Did it make sense?”

Ehm, in relation to the thing we picked up him up for or?

Yes but also if it make sense to investigate and if you think that you don’t understand the question, and if you need further instructions?

I do not know if I understand the question but the guy we just picked up, he did not need help so ehm… and since he had no problem walking… Then I do not know what to answer. But it makes good enough sense because he could have had a lesser mobility. But if it is a question about if people ask for help, even though we asses that people can walk, or is it the other way around? Is it like that?

“Yes but also if it make sense to investigate the mobility and if you under the question and know how to answer when we ask you “how much help did the patient need to be mobilized to the ambulance trolley?”

“ Yes that I understand”.

“Okay good, and the different answer categories are fine?”

“Yes, yes.”

“Okay, well I think that was what we needed.”

Interview five, date 17/2-2020, time 09:19

Position: Paramedic

Experience: 11 years

“ What is your understanding of the mobility question: How much help did the patient need to be mobilized to the ambulance trolley?”

“Ehm, I think I understand it completely.”

Yes, and you think that it made sense?”

“Yes, I think so.”

“Okay and what about the different answer categories?”

“Yes they are sufficient I think”

Interview six, date 17/2-2020, time 11:20

Position: paramedic

Experience: 11 years

“ What is your understanding of the mobility question: How much help did the patient need to be mobilized to the ambulance trolley?”

“I am pretty good at that”

“Yes and what do you think about the different answer categories?”

“The answer categories?”

“Yes a little, some, moderate and a lot?”

“ ehm, it something I have worked with before”

“No, does the questions make sense? Does it make sense to investigate? And do the answer categories fit? Do you think that there are lacking answer categories so a score can be given in relation to the patient you just had?”

“Ehm, what I think is, is that there are really many, when we arrive, then people are bedridden to some sort of degrees, and therefore it perhaps makes good sense to ask to the habitual state. But I can follow the idea that it is I an exiting project in relation to mortality. That I can follow”.

“Okay”

And in that way, then it makes good sense”

“And do you think that there were some answer categories that were lacking, perhaps I relation to other situations?”

“Not really”

“Okay so it was easy to fit the patient into one of the categories?”

“yes”.

Interviewer: Stine

Interview seven, date 14/2-2020

Position: Trainee

Experience: 1 year and 2 months

"What is your understanding of the mobility question: How much help did the patient need to be mobilized to the ambulance trolley?"

"I understand it as how much help the patient needs to be get onto the ambulance trolley and how much help we should offer. It is full support or does she only need a hand."

"How do you assess that?"

"On this trip it was the ambulance technician assessment to just give a hand"

"Does it make sense to ask the question like this?"

"I think it makes sense".

Interview eight, date 14/2-2020

Position: Paramedic

Experience: 12 years

"What is your understanding of the mobility question: How much help did the patient need to be mobilized to the ambulance trolley?"

"I think that it makes sense with answer categories that there were. It is a fine understanding"

Was there anything you thought could have been a better way to investigate the patient's mobility?

"No. You came with four options, and I think they fit really well for our patients".

Interview nine, date 14/2-2020

Position: Paramedic

Experience: 10 years

"What is your understanding of the mobility question: How much help did the patient need to be mobilized to the ambulance trolley?"

"I think that I understand it and I think it makes good sense. Maby there could be made an option for further elaboration of the purpose. It would make it easier for me to understand what it is supposed

to be used for. A little more background knowledge about what the project should end with, and what the purpose is. Because then, as I said, it would be easier for me to give a relevant answer."
